# Performance evaluation of two interventional fluoroscope suites for cardiovascular imaging

**DOI:** 10.1002/acm2.13741

**Published:** 2022-08-11

**Authors:** Gregory Anthony, Yun Liang, Xuandong Zhao

**Affiliations:** ^1^ Department of Radiology and Imaging Sciences Indiana University School of Medicine Indianapolis Indiana USA; ^2^ Krannert Cardiovascular Research Center Indiana University School of Medicine/IU Health Cardiovascular Institute Indianapolis Indiana USA

**Keywords:** fluoroscopy, image quality, radiation dose

## Abstract

Interventional cardiology involves catheter‐based treatment of heart disease, generally through fluoroscopically guided interventional procedures. Patients can be subject to considerable radiation dose due to prolonged fluoroscopy time and radiographic exposure, and therefore efforts to minimize patient dose should always be undertaken. Developing standardized, effective quality control programs for these systems is a difficult task owing to cross‐vendor differences and automated control of imaging protocols. Furthermore, analyses of radiation dose should be performed in the context of its associated effects on image quality.

The aim of the study is to investigate radiation dose and image quality in two fluoroscopic systems used for interventional cardiology procedures. Image quality was assessed in terms of spatial resolution and modulation transfer function, signal‐to‐noise and contrast‐to‐noise ratios, and spatial–temporal resolution of fluoroscopy and cineradiography images with phantoms simulating various patient thicknesses under routine cardiology protocols. The entrance air kerma (or air kerma rate) was measured and used to estimate entrance surface dose (or dose rate) in the phantoms.

## 1 INTRODUCTION

Fluoroscopically guided interventions continue to see increased use in healthcare due to the growing prevalence of complex interventional procedures, such as transcatheter aortic valve replacement and transjugular intrahepatic portosystemic shunt placement. Although beneficial in many aspects of patient care, these procedures can incur significant radiation‐related detriments, including both stochastic (e.g., cancer induction) and deterministic (e.g., skin reactions) effects. In order to mitigate this risk and improve quality of patient care, efforts should be made to minimize radiation dose to the patient and staff and optimize image quality to facilitate economization of dose and reduce beam‐on time. Indeed, many manufacturers of interventional fluoroscopic devices have made vast improvements to dose reduction techniques, image processing, and streamlined workflow. However, there are currently no universal criteria used for evaluation of such systems. Furthermore, differences in image processing, automatic exposure control (AEC) function, and system design between vendors render any objective assessments or comparisons virtually impossible.

Several quality control metrics, such as those introduced by Goode et al.,^1^ have shown promise in characterizing and tracking system performance across radiology departments and over time. That study examined a fleet of fluoroscopes from a single vendor, however. Other studies have compared the performance of different vendors’ fluoroscopy systems, but these have focused primarily on radiation dose reduction as a figure of merit, with image quality assessment being largely qualitative.^2^, ^3^

In this study, we performed an evaluation of image quality and radiation dose between two state‐of‐the‐art interventional fluoroscopy systems. Cardiac imaging protocols were tested on both systems, using default vendor‐provided settings. Through this assessment, we hope to provide a qualitative and quantitative characterization of these systems using reproducible and easy‐to‐implement tests, as well as develop a foundation of performance metrics for use in routine quality control.

## 2 MATERIALS AND METHODS

### 2.1 Fluoroscopy equipment, phantoms, and dosimeters

Image data were acquired with two interventional fluoroscopes: one Siemens Artis Q with CARE+CLEAR (Siemens Healthineers, Forchheim, Germany) and one Philips Azurion with ClarityIQ (Philips Healthcare, Best, The Netherlands). CARE is a dose reduction package utilizing five‐parameter AEC, and CLEAR is an image processing feature that serves to reduce noise, increase image sharpness, and compensate for motion artifacts.^4^, ^5^ ClarityIQ is an image processing feature, the algorithm of which improves image quality at low dose rates using automatic motion control and compensation, spatial and temporal noise reduction, and image enhancement with a multiresolution image decomposition approach.^6^, ^7^ The Artis Q system was manufactured in January 2020 and installed in July 2020. The Azurion system was manufactured in October 2018 and installed in July 2020. The Artis Q system is utilized clinically as a dedicated cardiac system, whereas the Azurion system is multipurpose. Both systems were operated using the default clinical cardiac imaging protocols from each institution: Coronary FL Card (‐) for the Artis Q and Left Coronary Clarity Classic (Xper) low FL frame rate for the Azurion. The software versions for the two systems were VD‐11‐E and 1.2.3 for Artis Q and Azurion, respectively.

**FIGURE 1:**
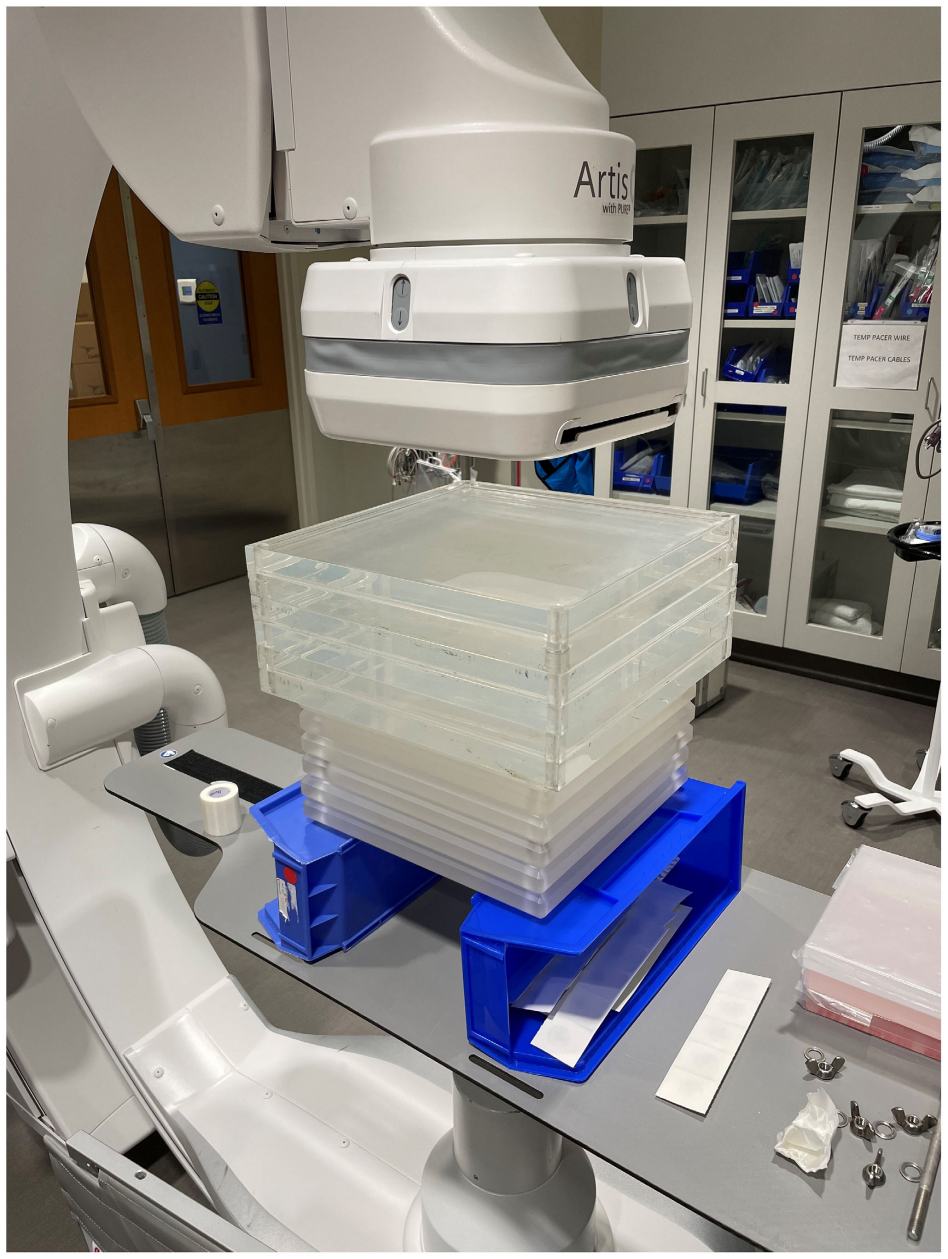
Polymethyl methacrylate (PMMA) slabs used for automatic exposure control (AEC) dosimetry measurements.

**FIGURE 2:**
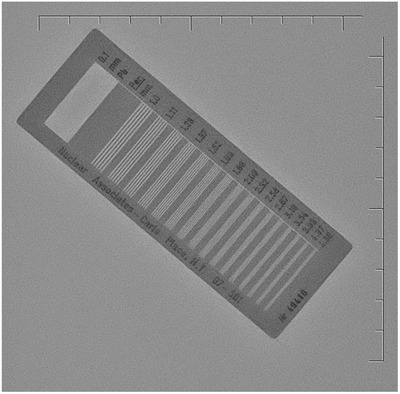
Line pair phantom used for spatial resolution measurements. The line pair phantom was affixed to the image receptor surface and exposed with three simulated patient thicknesses in the beam (6, 9, and 12 in. polymethyl methacrylate [PMMA]) to assess limiting spatial resolution.

**FIGURE 3:**
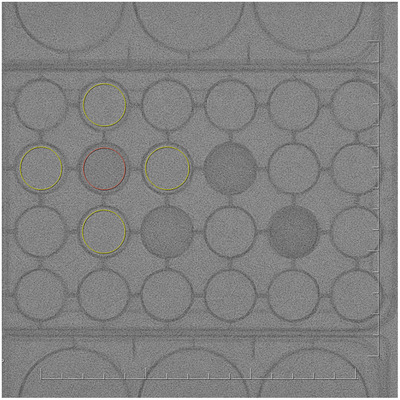
A 24‐well specimen plate filled with 1‐ml samples of iopamidol contrast (Isovue 370 with dilution factors of 80×, 40×, 20×, and 10×, from left to right). The plate was placed on top of simulated patient thicknesses of 6, 9, and 12 in. polymethyl methacrylate (PMMA). Contrast (red) and background (yellow) regions of interest (ROIs) were drawn for contrast‐to‐noise ratio (CNR) measurement.

A variety of phantoms were used for image quality and radiation dose evaluation. For AEC and dose testing, 1‐in.‐thick slabs of polymethyl methacrylate (PMMA) with 12 × 12‐ or 14 × 14‐in. areas were stacked in thicknesses of 2–15.5 in. (Figure 1). A line pair phantom with patterns ranging from 1.0 to 4.86 LP/mm (Nuclear Associates Model 07–501, SN: 49410, Carle Place, NY) was used for measurements of limiting spatial resolution (Figure 2). Modulation transfer function (MTF) calculations were performed using the edge of a 1‐mm‐thick copper (Cu) plate. Signal‐to‐noise ratio (SNR) and contrast‐to‐noise ratio (CNR) measurements were performed using three thicknesses of PMMA slabs, along with a 24‐well specimen plate (PN: 353047, Falcon, Corning, NY) filled with 1‐ml samples of iopamidol contrast (Isovue 370, Bracco Diagnostics, Princeton, NJ) diluted by factors of 10, 20, 40, and 80 for the CNR measurements (Figure 3). Finally, spatial–temporal resolution was assessed using an infusion catheter (20‐cm length, *V* = 0.29 ml, Terumo Surflo, Tokyo, Japan) filled with 12.3 mg/ml Isovue (30× dilution) affixed to the top of a motorized rotating spoke test tool (L‐629, Ludlum Measurements, Inc., Sweetwater, TX) with three thicknesses of PMMA slabs. The rotating test tool moved at a rate of 0.5 Hz, equating to a maximum spoke velocity of 21.4 cm/s at the outer edge of the rotating circle.

Air kerma rate measurements were performed using a transparent ion chamber and dosimeter (Unfors RaySafe Xi, Billdal, Sweden). Half‐value layer (HVL) measurements were also performed with a solid‐state dosimeter and digitizer (RadCal AGMS‐D+, Accu‐Gold+, Monrovia, CA) placed outside the AEC measurement region. Proper placement was verified with repeated measurements to ensure that fluoroscope output did not change as a result of the presence of the solid‐state dosimeter.

### 2.2 Image and dose data collection and analysis

Dosimetry data were acquired for pulsed fluoroscopy using the transparent ion chamber at the IEC patient entrance reference point (PERP), 15 cm from C‐arm isocenter toward the X‐ray tube. A range of patient thicknesses were simulated using 2–15.5 in. PMMA. For all simulated patient thicknesses, air kerma rate (mGy/min) and air kerma per pulse (mGy) were measured at the PERP. Table height remained constant over all PMMA thicknesses tested. Measurements were corrected for system differences in focal spot to PERP distance. Half value layer measurements were made concomitantly using the solid‐state dosimeter. Both systems were evaluated at the same pulse rate (7.5 pps) with the most commonly used clinical fluoroscopy setting. SID was set to the maximum value for both systems (120 cm). Patient entrance surface dose for a patient with skin located at the PERP was estimated using backscatter factors and air‐tissue conversion factors as documented by Benmakhlouf et al.^8^

For SNR evaluation, flat field images of three simulated patient thicknesses (6, 9, and 12 in. PMMA) were acquired in fluoro mode at fields of view (FOVs) of 25 (27), 20 (19), and 16 (15) cm for the Artis Q (Azurion) system. The last 15 frames (2 s) of a 5–10‐s exposure were saved in DICOM format. Images were imported into Matlab (R2021a MathWorks, Natick, MA) for analysis using a custom‐scripted workflow. An average SNR value was determined for each simulated thickness by measuring the mean signal and standard deviation in a central region of interest (ROI) comprising approximately 10% of the full FOV. Image smoothing and edge enhancement settings were left at the default level for both systems. CNR evaluation was performed similarly, using simulated patient thicknesses of 6, 9, and 12 in. PMMA and the 24‐well specimen plate described earlier. Each contrast‐filled well and the four wells adjacent to it were used to compute average contrast and noise standard deviation values (Figure 3). The last 15 frames (2 s) of the fluoro loop were used to compute an average CNR for each simulated thickness. Additionally, the dose efficiency for each system and thickness was computed as

(1) $$E_{SNR}=\frac{SNR}{\sqrt{K_{air}}}$$

(2) $$\;E_{CNR}=\frac{CNR}{\sqrt{K_{air}}}$$

using the average measured SNR or CNR for a given exposure setting and the square root of the measured air kerma rate $\sqrt{K_{air}}$ at the PERP.

The line pair phantom was affixed to the image receptor surface and exposed with three simulated patient thicknesses in the beam (6, 9, and 12 in. PMMA) to assess limiting spatial resolution. The maximum resolvable spatial resolution in LP/mm was determined by a visual inspection of fluoro mode images.

The system MTF was determined for each fluoroscope using a 9‐in. PMMA simulated thickness and a Cu sheet positioned such that a slanted edge was created in the middle of the X‐ray field. Fluoro loops were acquired using comparable FOVs (25/27, 20/19, and 16/15 cm for Artis Q/Azurion, respectively) for at least 5 s, and the last 15 frames (2 s) of data were recorded. These frames were averaged and used to compute the presampled MTF with an ImageJ plugin for slanted edge MTF calculation.^9^ The MTF was calculated using 128 pixels across the edge, which produced an effective balance between detail/accuracy of the curve and noise.

**FIGURE 4:**
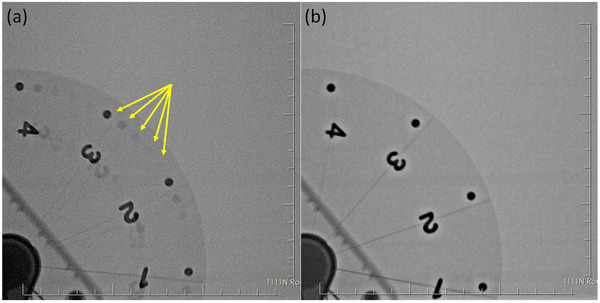
Fluoroscopy (a) and cineradiography (b) images of rotating spoke phantom used to assess temporal resolution/motion blur. Images were acquired at 25‐cm field of view (FOV) on the Artis Q system with 6 in polymethyl methacrylate (PMMA) attenuation. Yellow arrows indicate “ghost” objects due to recursive filtering of fluoroscopy images.

Pulsed fluoroscopy and digital cineradiography acquisitions of the rotating spoke phantom with the iodine‐filled catheter were performed using the clinical cardiac protocols for simulated patient thicknesses of 6, 9, and 12 in. PMMA. Cine and fluoro mode images were recorded at comparable FOVs (25/27, 20/19, and 16/15 cm for Artis Q/Azurion, respectively) and played off‐line for visual inspection and estimation of motion blur (Figure 4). The thinnest resolvable phantom spoke in fluoro and cine modes was recorded for each FOV and thickness. The full‐width at half‐maximum (FWHM) of the contrast catheter was also measured at three different radii (10‐, 25‐, and 40‐mm inward from the disk edge) in the cine mode images, representing three distinct velocities on the rotating test tool (18.3, 13.5, and 8.8 cm/s), and compared with the measured catheter FWHM in stationary images (i.e., test tool not rotating) to estimate motion‐induced blurring.

Recursive filtering of sequential images is a common noise reduction technique in fluoroscopy. This process was evaluated by recording signal intensity in a fixed ROI over multiple frames. A lead marker was present in the ROI during the first frame, generating a background signal recovery over successive frame‐averaged images. ROI data over five frames (in which no objects other than the lead marker passed in front of the ROI) were fit to the following equation:

(3) $$y=A+B\left(1-e^{-Cx}\right)$$

where *A* is the initial signal intensity in the lead marker, *B* is the contrast between the lead marker and background, and *C* is the rate at which residual signal from the lead marker disappears. For a recursively filtered image, the intensity of which is given by

(4) $$\;I_{n}=\alpha S_{n}+\left(1-\alpha \right)I_{n-1}$$

where $I_{n}$ is the displayed image intensity of frame *n*, $S_{n}$ is the measured signal of frame *n*, and α is a weighting factor between 0 and 1,^10^ it can be shown that the factor α is equivalent to $1-e^{-C}$, using the fitted value of *C* from Equation (3). Values for α were calculated thusly and reported as the average over multiple fittings using three separate sets of five frames.

## 3 RESULTS

### 3.1 Dosimetry

**FIGURE 5:**
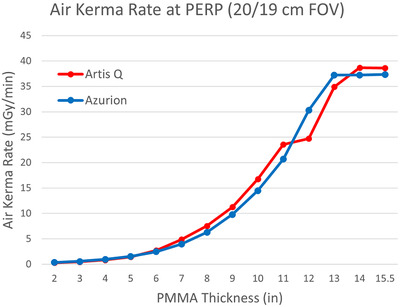
Air kerma rate in mGy/min using intermediate (20/19 cm) field of view (FOV) for varying thicknesses of polymethyl methacrylate (PMMA). The two systems had distinct automatic exposure control (AEC) functions but still produced comparable air kerma rates over the range of patient thicknesses simulated.

**FIGURE 6:**
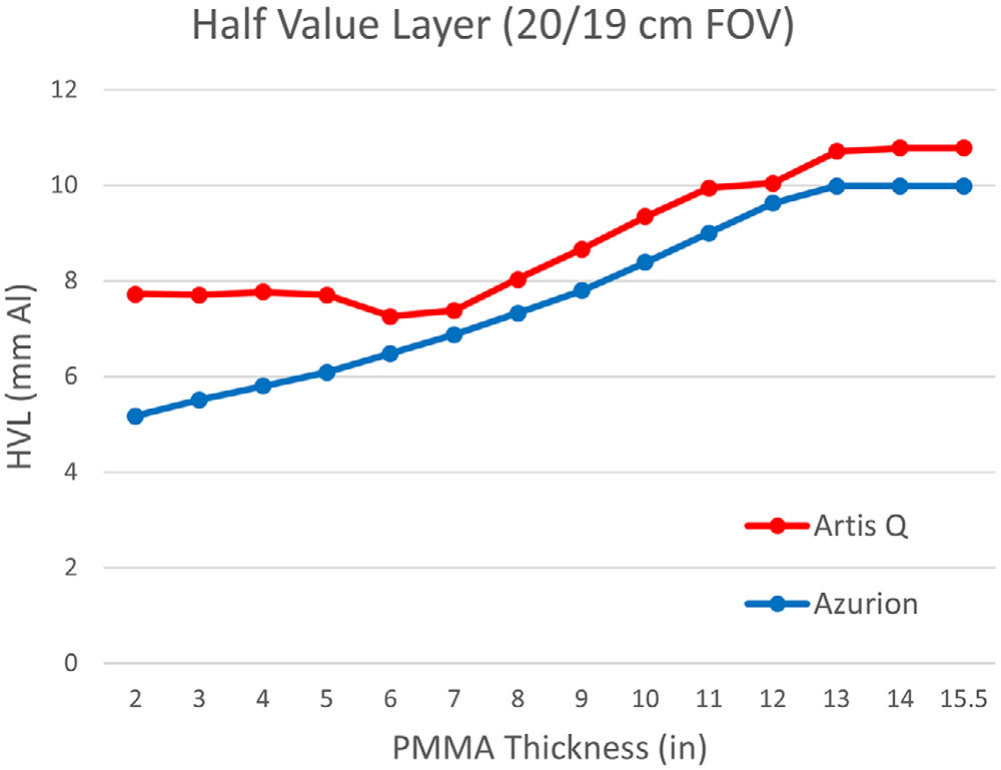
Half‐value layer in mm Al for varying levels of phantom attenuation. The Azurion system half‐value layer (HVL) changed continually as a result of kVp adjustment with phantom thickness. The Artis Q system maintained a consistent beam quality for lower phantom thicknesses through the use of dynamic beam filtering.

**FIGURE 7:**
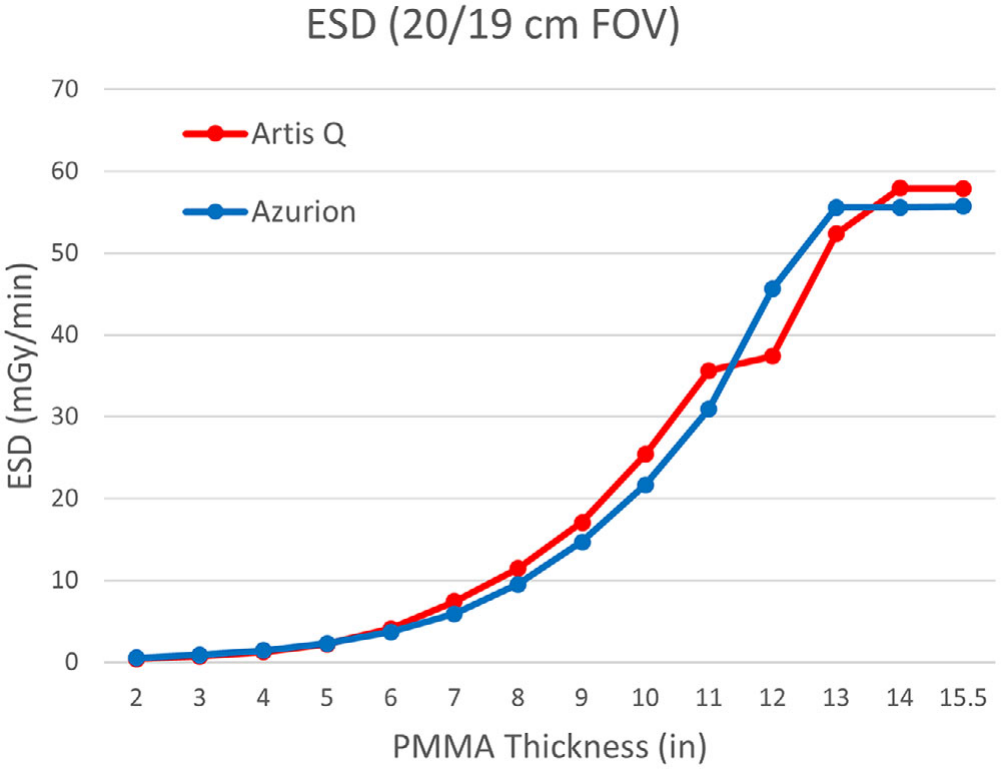
Estimated entrance skin dose rate in mGy/min using intermediate (20/19 cm) field of view (FOV) for varying levels of phantom attenuation. The skin entrance of the simulated patient was assumed to be at the patient entrance reference point (PERP) (15 cm below device isocenter).

Both fluoroscopy systems indicated reference air kerma rates within 20% of measured values. The two AEC systems caused similar changes in air kerma as phantom thickness was added (Figure 5). Air kerma at the same distance from the X‐ray source (after correction for differences in source‐to‐PERP distance) was comparable for both systems, with Artis Q using a slightly higher air kerma rate for intermediate patient sizes and a slightly lower air kerma rate for large patient sizes. This was the result of a more complex AEC function for the Artis Q system, which involved dynamic focal spot and filter selection in addition to tube voltage and current modulation.^4^, ^11^ The Azurion system produced a consistently lower half value layer that continually decreased with smaller phantom thicknesses, whereas the Artis Q system maintained a constant HVL below thicknesses of 6–7 in. of PMMA (Figure 6). The air kerma rate and HVL curves for the Artis Q system also demonstrate a discontinuity between 11‐ and 12‐in. PMMA, corresponding to a transition to a larger focal spot size once the tube load is maximized. This resulted in a reduced air kerma rate for the largest thicknesses with the Artis Q system. Estimated entrance skin doses varied similarly to air kerma values as phantom thickness increased (Figure 7).

### 3.2 SNR dose efficiency

**FIGURE 8:**
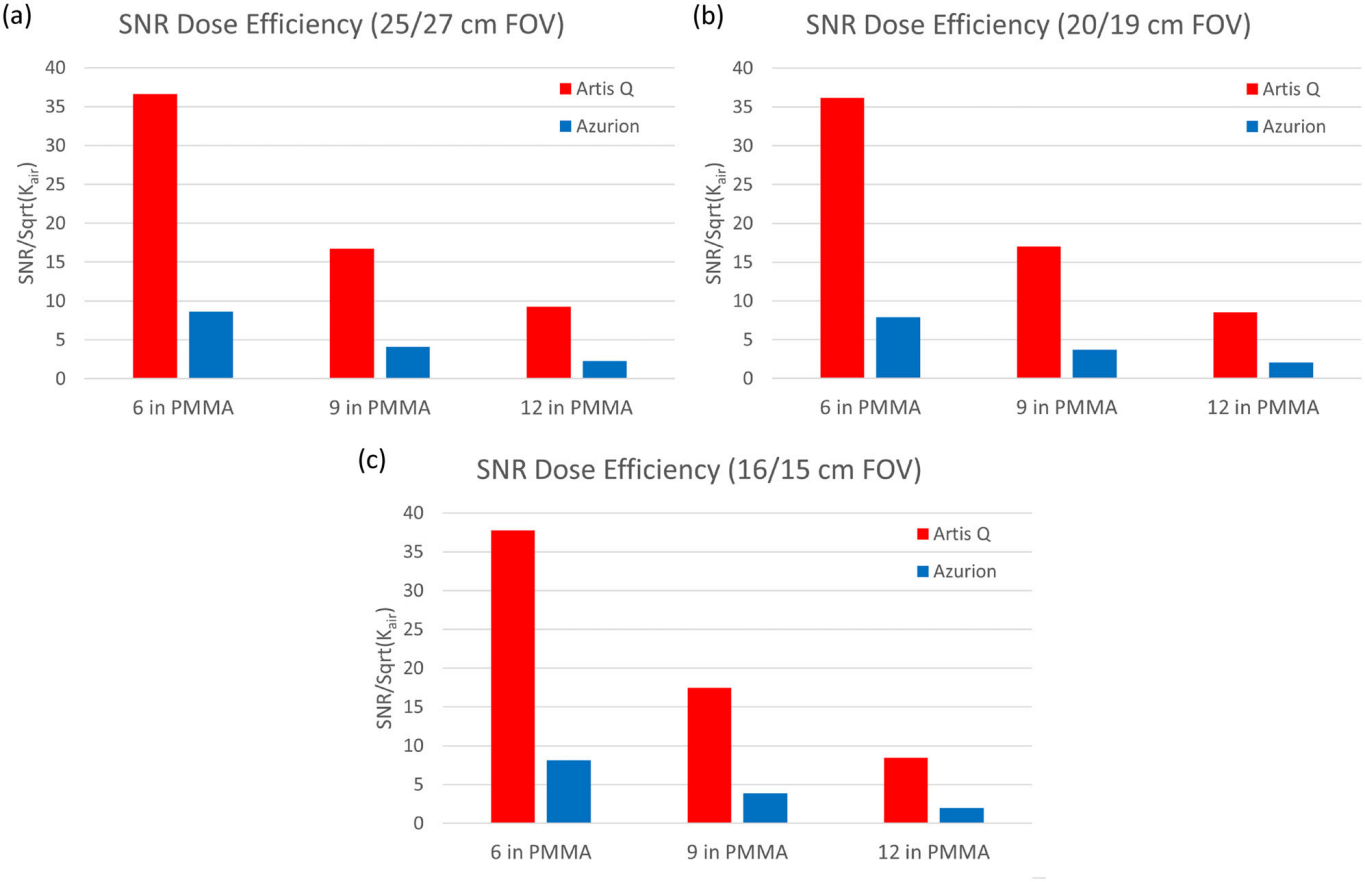
Signal‐to‐noise ratio (SNR) dose efficiency for three simulated patient thicknesses at (a) 25/27‐cm field of view (FOV), (b) 20/19‐cm FOV, and (c) 16/15‐cm FOV. The Artis Q system exhibited consistently lower image noise per unit air kerma rate across all thicknesses.

The SNR dose efficiency (i.e., considering image noise, but not contrast, as a function of air kerma rate) was compared for the three largest available FOVs on the Artis Q system (25, 20, and 16 cm) and the most comparable FOVs on the Azurion system (27, 19, and 15 cm). SNR dose efficiency for the Artis Q system was 4.1–4.6 times greater than that of the Azurion system. (Figure 8). This ratio was consistent across FOVs and simulated patient thicknesses. SNR and air kerma rates both increased with smaller FOVs, leading to a relatively constant SNR dose efficiency over all FOVs tested.

### 3.3 CNR dose efficiency

**FIGURE 9:**
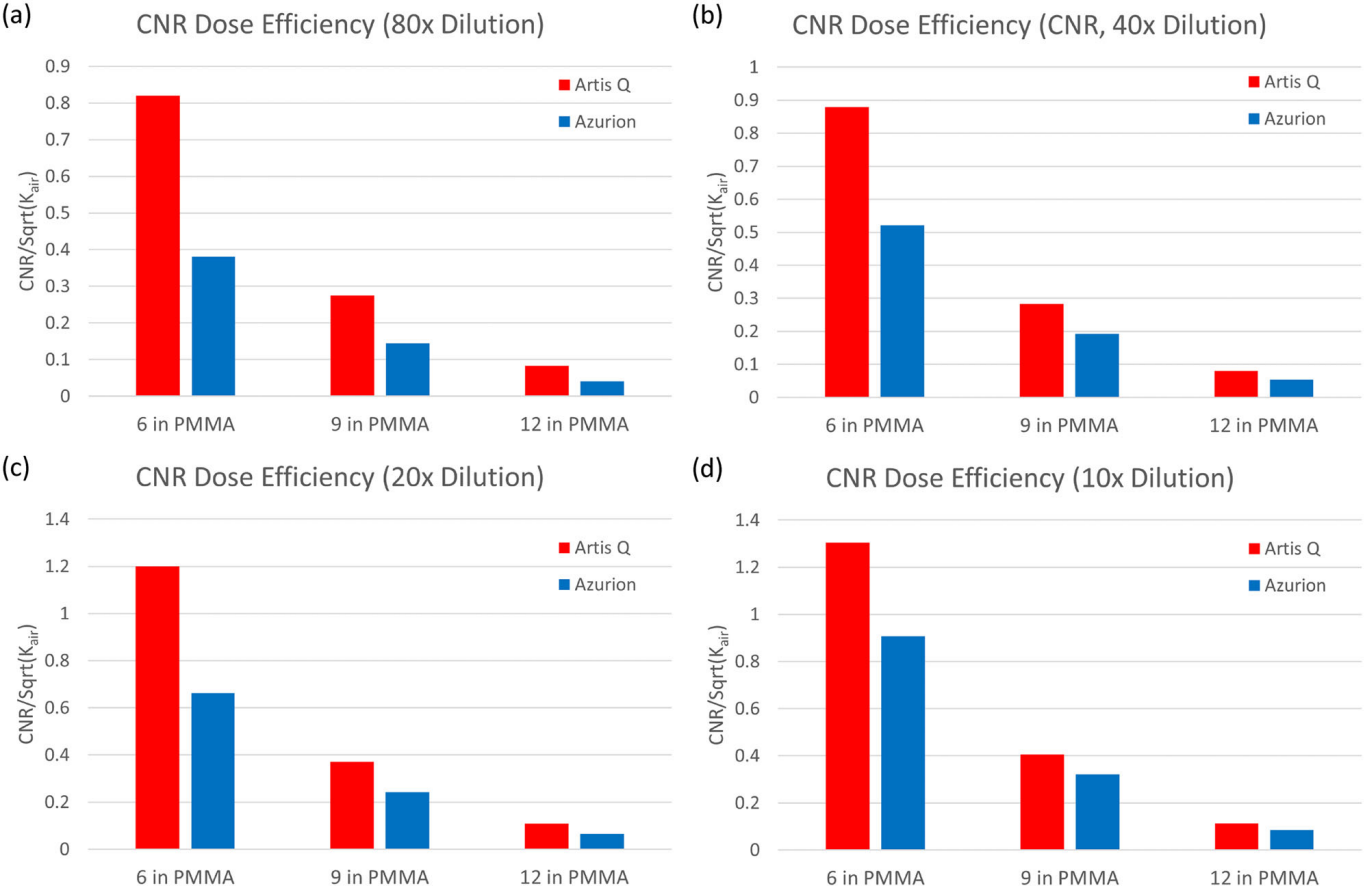
Contrast‐to‐noise ratio (CNR) dose efficiency for three simulated patient thicknesses with (a) 80× dilution (4.625 mg/ml), (b) 40× dilution (9.25 mg/ml), (c) 20× dilution (18.5 mg/ml), and (d) 10× dilution (37 mg/ml) of Isovue 370. The Artis Q system exhibited consistently greater CNR per unit air kerma rate across all thicknesses, with a greater difference in performance for small simulated patient sizes.

When comparing CNR per unit air kerma rate for the iodine concentrations listed earlier, the Artis Q demonstrated a 65% improvement in dose efficiency over the Azurion on average (range: 26%–115%; Figure 9). This improvement in CNR performance was greatest for the 6‐in. PMMA thickness. However, when comparing contrast alone (normalized to background intensity), the Azurion system produced 88% greater contrast on average (range: 32%–166%). These observed trends were similar across all iodine concentrations tested.

### 3.4 Limiting spatial resolution

The maximum resolvable spatial resolution was comparable for both systems. The Artis Q images exhibited some loss of spatial resolution for small and intermediate patient sizes, possibly due to greater image smoothing to offset noise. At similar air kerma rates, Azurion images were visually sharper than Artis Q images. Table 1 gives the maximum resolvable spatial resolution for both systems at each magnification mode and phantom thickness.

**TABLE 1:** Maximum resolvable spatial resolution inline pairs per millimeter (LP/mm) for each system at three phantom thicknesses and three magnification levels (fields of view [FOVs])

Artis Q		Azurion	
FOV (cm)	Max spatial resolution (LP/mm)	FOV (cm)	Max spatial resolution (LP/mm)
6 in. PMMA			
25	2.32	27	2.32
20	2.58	19	2.87
16	2.58	15	2.87
9 in. PMMA			
25	2.09	27	2.09
20	2.32	19	2.32
16	2.32	15	2.58
12 in. PMMA			
25	1.88	27	1.52
20	2.09	19	2.09
16	2.09	15	2.32

**^Abbreviation:^ :**PMMA, polymethyl methacrylate.

### 3.5 Modulation transfer function

**FIGURE 10:**
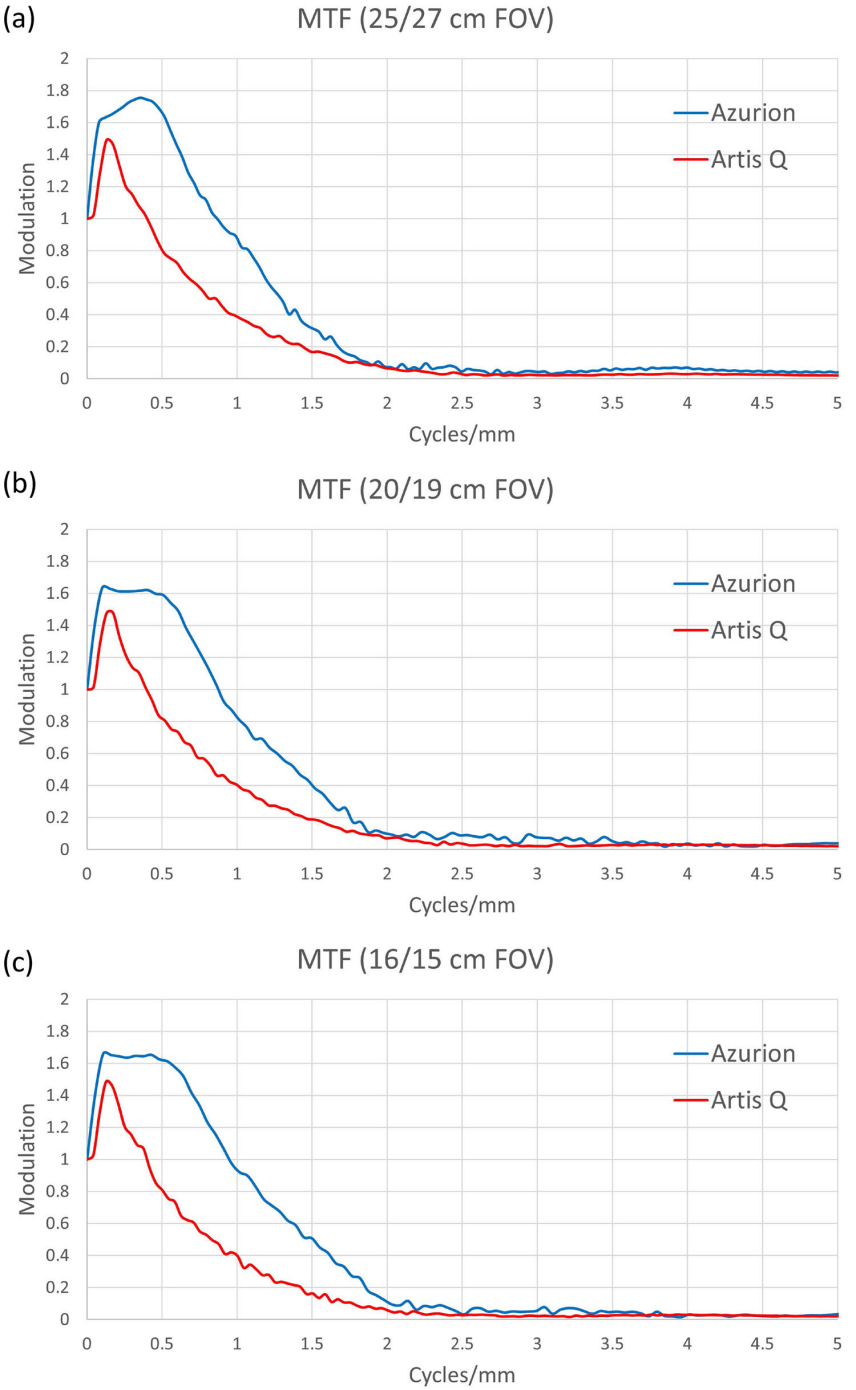
Modulation transfer function (MTF) curves for Azurion and Artis Q systems at (a) 25/27‐cm field of view (FOV), (b) 20/19‐cm FOV, and (c) 16/15‐cm FOV. Elevation of the curves above unity indicates edge‐enhancement processing effects. The Azurion demonstrated a greater degree of edge enhancement, but both systems exhibited similar limiting spatial resolution between 1.8 and 2.0 cycles/mm.

MTF curves for both systems showed some degree of edge enhancement, indicated by increases in the MTF above the unity value assigned to 0 cycles/mm (Figure 10). The Azurion system MTF curve suggested stronger edge enhancement, with a maximum modulation of 1.75 at 0.36 cycles/mm. This resulted in improved spatial resolution performance by the Azurion system, as indicated by the 50% MTF values in Table 2. Limiting spatial resolution (where MTF drops to 10%) was approximately 1.8–1.9 cycles/mm for both systems, with some improvement at smaller FOVs for the Azurion system. For the Artis Q system, the MTF remained constant as FOV was decreased. This was due to the reduced matrix size of output images at smaller FOVs, which maintained an effectively constant pixel size. The Azurion image matrix size was also reduced upon export, but not in proportion with the FOV, leading to a range of exported pixel sizes (197–146 μm). This led to an underestimation of the Artis Q MTF when compared with that of the Azurion at smaller FOVs.

**TABLE 2:** 50% and 10% modulation transfer function (MTF) values in cycles per millimeter (cyc/mm) for each system at 9 in. polymethyl methacrylate (PMMA) thickness and three magnification levels (fields of view [FOVs])

Artis Q			Azurion		
FOV (cm)	50% MTF (cyc/mm)	10% MTF (cyc/mm)	FOV (cm)	50% MTF (cyc/mm)	10% MTF (cyc/mm)
25	0.86	1.80	27	1.27	1.86
20	0.82	1.81	19	1.37	1.87
16	0.84	1.75	15	1.50	2.03

### 3.6 Spatial–temporal resolution

**FIGURE 11:**
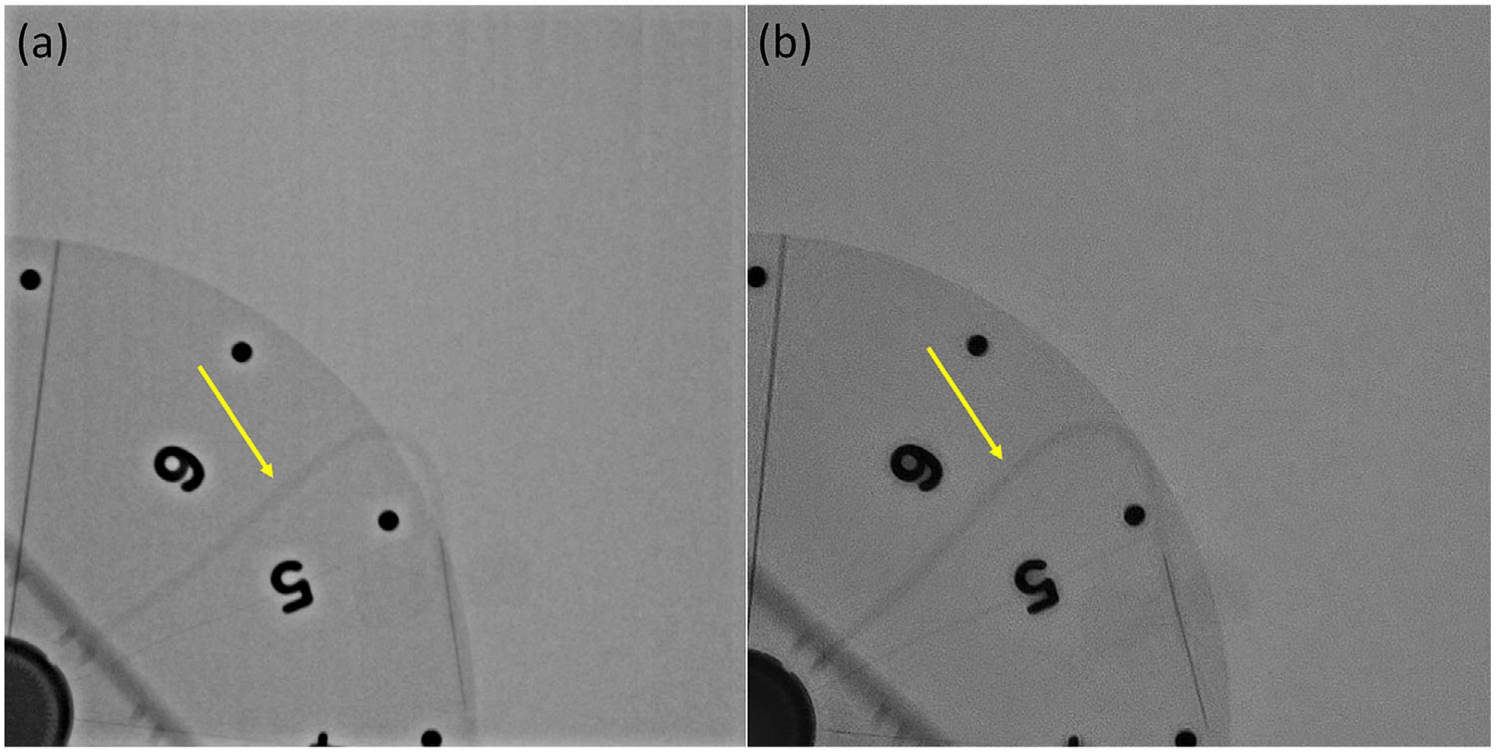
Siemens Artis Q (a) and Philips Azurion (b) cine images of rotating spoke phantom at 25/27‐cm field of view (FOV) with attenuation of 6 in. polymethyl methacrylate (PMMA). Contrary to fluoro mode, edge enhancement effects are more pronounced in the Artis Q images. Yellow arrows indicate iodine‐filled infusion catheter.

The thinnest resolvable phantom spoke in cineradiography images was identical for both systems (Table 3). Motion‐induced blurring of the catheter in cineradiography images was not visually apparent for either system. Relative to the static catheter FWHM, measured widths of the moving catheter ranged from 82% to 110% and from 85% to 121% of static width for the Artis Q and Azurion, respectively. Uncertainty (coefficient of variation) in measurements of the static catheter width across FOVs, attenuation levels, and radii were 6.6% and 5.6% for the Artis Q and Azurion, respectively. Errors in width measurement from cine images with motion were primarily due to excessive noise and low conspicuity of the contrast catheter, particularly for 12 in. PMMA attenuation. No difference in catheter width was observed between different FOVs or catheter radii. Fluoroscopy images also resolved the same number of wheel spokes for both systems, although phantom spoke and catheter conspicuity were notably decreased due to lower tube output and lack of motion correction in this mode. Qualitatively, cineradiography images from the Azurion system appeared to have less uniform noise properties, whereas Artis Q images had more noticeable edge enhancement “halo” artifacts (Figure 11).

**TABLE 3:** Thinnest resolvable spoke from rotating phantom (1 = thickest, 6 = thinnest) for each system at three phantom thicknesses

	Cine mode		Fluoro mode	
	Artis Q	Azurion	Artis Q	Azurion
6 in. PMMA	5	5	4	4
9 in. PMMA	5	5	3	3
12 in. PMMA	3	3	1	1

**^Abbreviation:^ :**PMMA, polymethyl methacrylate.

The exponential model described by Equation (3) was an excellent fit to the fluoro image data (*R* ^2^ ≥ 0.99). The coefficient α was comparable across attenuation levels and FOVs for both systems. The Artis Q exhibited a smaller degree of image lag from recursive filtering, with an α of 0.829 (range: 0.792–0.859), compared with an α of 0.704 (range: 0.669–0.727) for the Azurion system. Both systems were operating at 7.5 frames/s.

## 4 DISCUSSION

In this study, we characterized two interventional cardiovascular fluoroscopes in terms of image quality and radiation dose. Both the Artis Q and Azurion systems were operating in a low‐dose mode, which was the commonly used default setting in their routine clinical operation. From a dose perspective, the two systems performed comparably over the range of phantom thicknesses tested. This contrasts with a previous study from Trunz et al., which found significantly lower dose rates in Philips Allura systems with Clarity IQ than in an Artis Q system with PURE and CLEAR.^3^ The Azurion system in the current study is a more recent model than the Allura but also had Clarity IQ installed. The Artis Q system in the current study had CARE+CLEAR installed, rather than CLEAR, which may account for the more comparable dose performance with the Azurion system. As dose rate is readily adjustable for modern fluoroscopy systems and could affect image quality, it is necessary to relate dose quantification with resultant image quality. In this study, the SNR, CNR, MTF, and spatial and spatial–temporal resolution were thus evaluated in parallel with dose in both systems.

One notable difference between the systems is how the beam HVL is modulated with phantom attenuation. Although the Azurion system appears to compensate for added attenuation by increasing kVp and mAs continually, the Artis Q system maintains a minimum kVp of 70 while increasing mAs up to approximately 6 in. of PMMA. Beyond this thickness, the mAs is fixed at a maximum value of 240 while kVp increases. This accounts for the harder, constant beam quality observed at low phantom thicknesses for the Artis Q system. Additionally, the Artis Q system features a dynamic filtering approach to AEC (Johann Seissl), which changes the beam filtration depending on attenuation.^12^, ^13^ The decrease in HVL at approximately 6 in. of PMMA thickness (Figure 6) is the result of this dynamic filtering, as a thicker copper filter is swapped for a thinner one to allow adequate photon fluence beyond this thickness. On the other hand, the Azurion system maintains a fixed copper filter thickness for a given imaging protocol, and its HVL is therefore driven purely by kVp. The Artis Q's unique method of technique modulation likely explains the slight increase in air kerma over the Azurion system for most intermediate phantom sizes above 6 cm, as the thinner filter is utilized. Both systems produce comparable air kermas for very small phantoms due to the added filtration at smaller thickness for the Artis Q. However, the Artis Q system's dynamic focal spot also causes a discontinuity in the HVL and air kerma rate curves that reduces the air kerma rate for equivalent thicknesses above 11–12 in. PMMA. This results in similar maximum air kerma rates for both systems, despite the Artis Q's higher maximum tube voltage (125 kVp). The disparate beam qualities of the two systems did not create any large differences in entrance skin dose that were not explainable by air kerma differences.

When considering noise properties alone (without contrast), dose efficiency for the Artis Q system was higher than the Azurion system by roughly a factor of four on average. Considering the similar air kerma rate findings, this superior efficiency is reflective of the greater noise reduction in the processed images from the Artis Q system. The noise reduction likely involves some degree of spatial filtering (i.e., image smoothing), as evidenced by the differences in MTF curves and visual sharpness between the two sets of images. However, limiting spatial resolution was similar for both systems. This is likely a result of the noise reduction algorithms of CLEAR^5^ and ClarityIQ,^7^ both of which identify image signal structures at multiple spatial resolution levels, then smooth only those parts of the image that do not contain signal features. The increase in SNR at smaller phantom sizes was likely due to downsampling of the images’ matrix size upon export, which would reduce noise through interpolation. Although matrix sizes were identical for the large (25/27 cm) FOV, the Azurion SNR is underestimated at smaller FOVs due to unequal downsampling schemes between the systems.

Contrast‐to‐noise ratio is likely a better indicator of image quality and utility in the interventional setting and will depend on beam quality as well as quantum noise. Thus, a CNR dose efficiency metric was computed for each system. The difference in CNR dose efficiency between the two systems (65%) was not as large as the difference in SNR dose efficiency (436%), owing to the improved contrast produced by the Azurion system. However, this was not sufficient to offset the lower noise levels seen in the Artis Q images. It should be noted that, in the strictest sense, dose efficiency metrics for SNR and CNR apply to raw, unprocessed images because post‐processing can alter these metrics for a given air kerma rate. Therefore, the dose efficiency values listed here refer to the default processing settings of each system; although they do provide a meaningful indication of clinical image quality for a given air kerma rate and application setting, they do not completely characterize these systems.

Characterizing the spatial resolution of interventional fluoroscopy systems is highly dependent on image processing settings, such as edge‐enhancement or smoothing levels. The limiting resolution at the detector face was comparable, as expected due to the similar pixel pitch for the two systems (197 and 184 μm for Azurion and Artis Q, respectively). A more complete characterization of spatial resolution performance via the calculation of MTF curves revealed large differences in edge enhancement between the two systems, even at default processing settings. However, these results are limited by the downsampling of exported images, which was not consistent between the two systems. As with dose and image noise settings, the level of edge enhancement or smoothing can be programmed based on user preference.

One important aspect of interventional fluoroscopy performance is compensation for motion in the image field. This is determined by the duration of each pulse, the degree of frame averaging, and motion correction algorithms employed by the system. Image intensifier–based systems are subject to image lag and ghosting due to the carryover of image charge generated by previous X‐ray exposures into subsequent image frames. However, modern flat‐panel systems markedly reduce the impact of these effects,^14^, ^15^ such that apparent image lag is primarily related to temporal image processing. The present study aimed to characterize fluoro and cine mode image quality in the presence of motion by visualizing thin, rotating phantom objects and contrast‐filled catheters. Figure 4 demonstrates the difference in motion compensation applied to fluoroscopy mode images and cineradiography images. In general, cineradiography utilizes a higher output and motion correction algorithms that compensate for small movements and limit frame averaging to low‐contrast, static portions of the image. The result is an image with very little blurring or noise that preserves the useful image structure information. This can be seen in the fluoroscopy and cine mode results from the spoke phantom and catheter: In cine mode images, more spokes were clearly resolved and the catheter was more conspicuous, whereas fluoroscopy images showed recursive filtering artifacts (“ghost” images of previous frames superimposed on the spoke wheel).

Measurements of vessel diameter to evaluate stenoses or aneurisms are common in coronary angiography, and thus a moving iodinated catheter was imaged to determine the degree to which motion confounds accurate diameter measurements in each system. These measurements were difficult due to high levels of image noise and the low visibility of the catheter at larger phantom thicknesses, but motion‐induced distortions of vessel width did not surpass 21%. In fluoro images, motion manifests primarily as residual signal in later frames due to recursive temporal filtering. The fluoro image analysis revealed that the Azurion system utilizes a lower value of α in its default recursive filter setting, which enables greater noise reduction at the cost of more perceived lag from persistence of previous frames.^10^ Due to the greater level of spatial smoothing applied by default to the Artis Q images, it is likely that less contribution is required from prior frames to achieve acceptable fluoro image noise, prompting selection of a larger value for α. Although both systems performed comparably in the number of visible spokes and catheter FWHM measurements for a given imaging mode, clear qualitative differences were seen between the cineradiography images. The Artis Q images contained notable edge‐enhancement artifacts manifesting as bright “halos” around high‐contrast objects such as the spoke wheel numbers (Figure 11a). The Azurion images appeared visually sharper overall but had less uniform noise properties (Figure 11b). It should be noted that cine mode image processing is separate from fluoroscopy mode image processing but can still be tailored by the user.

The goal of this study was to evaluate two interventional fluoroscopy systems from different vendors from an objective standpoint, considering both dose and image quality metrics. This study characterized dose at the PERP using a constant table height with varying attenuation, although many dose‐conscious interventional radiologists would likely maintain the patient as close to the image receptor as possible. Nonetheless, maintaining a constant table height regardless of patient size is a common practice in the interventional clinical setting. Furthermore, this experimental setup enabled quicker measurement and ensured that the observed changes in dose were due to changes in PMMA thickness, which is appropriate for a system‐wise comparison with identical SIDs (after correction for source‐to‐PERP distance differences). This study may not wholly represent the clinical performance of these two systems, being limited to phantom‐based evaluation of certain default clinical protocols and user settings. A more thorough characterization of these systems would also involve testing different fluoroscopy flavors, dose levels, and image processing schemes. Nonetheless, adjusting these parameters can make for a confusing analysis. For instance, a lower dose setting may increase image noise, but other post‐processing options can be enabled to offset or cancel this effect. This may in turn negatively affect the spatial resolution or conspicuity of small or low‐contrast objects. The performance of these systems is highly dependent on which of these features the users choose to implement. Therefore, this study aimed to evaluate common clinical protocols and default dose and image processing settings on both systems in order to perform an “out of the box” product analysis. It is also recognized that this work is based on highly processed images and standard phantoms. Qualitative evaluation of clinical images from interventional radiologists would further bolster the quantitative and qualitative results presented here. More advanced characterizations of system performance have also been described previously, including SNR evaluation using model observer frameworks^16^, ^17^ and calculations of spatiotemporal MTF from a moving slanted edge.^18^ However, these techniques require access to unprocessed data, extensive post‐processing and analysis, and some apparatus that are not readily obtainable. Although somewhat simplistic, the methods used in the current study are of low cost, easily reproducible, and provide a relatively comprehensive overall evaluation of the systems. In addition, these methods of SNR, CNR, and motion assessment align with recommendations put forth in the report of AAPM Task Group 272 on acceptance testing and evaluation of fluoroscopy imaging systems.^19^

## 5 CONCLUSION

This study demonstrated very similar performance from both systems in terms of radiation dose and spatial–temporal resolution performance. Image noise properties and the degree of edge enhancement were very different between the systems owing to differences in processing of the for‐presentation images. From an objective standpoint, both products’ default configurations are well‐matched in most respects, with the exception of the image noise/edge‐enhancement tradeoff. For the default post‐processing settings, the Artis Q system appears to be configured for greater image smoothing to improve CNR, whereas the Azurion applies stronger edge enhancement and recursive filtering to balance image sharpness and noise. The Azurion system's beam quality also produces greater overall contrast. Visual differences will likely lead to a preference of users for particular systems and hardware or software packages.

## CONFLICT OF INTEREST

The authors have no conflict of interest to declare.

## AUTHOR CONTRIBUTION

Gregory Anthony, Xuandong Zhao, and Yun Liang devised the concept and performed data acquisition. Gregory Anthony performed the data analysis and wrote the manuscript. Yun Liang and Xuandong Zhao also contributed to the interpretation of the data and refinement of the manuscript, as well as approval of the final version.
